# Acute Ostial Right Coronary Artery Occlusion During Valve Deployment of Transcatheter Aortic Valve Replacement Leading to Acute Right Ventricular Failure: A Perfect Storm and Successful Navigation

**DOI:** 10.7759/cureus.12373

**Published:** 2020-12-30

**Authors:** Udit Joshi, Padmaraj Duvvuri, Marco Barzallo, Sudhir Mungee

**Affiliations:** 1 Cardiology, University of Illinois College of Medicine at Peoria, Peoria, USA

**Keywords:** trans-catheter aortic valve replacement, right sided cardiogenic shock, acute right coronary artery occlusion, extracorporeal membrane oxygenation support, right ventricular assist device

## Abstract

Acute coronary obstruction is a relatively rare complication of transcatheter aortic valve replacement (TAVR). Left coronary ostial obstruction is much more common compared to right coronary occlusion due to its relatively lower ostial height from the aortic annulus. We present a case of acute ostial right coronary occlusion immediately upon deployment of a 29-mm Sapien 3 transcatheter aortic valve. The acute right coronary ostial occlusion manifested with ventricular fibrillation, acute right ventricular failure, and right-sided cardiogenic shock. The patient, after undergoing an initial unsuccessful attempt at percutaneous revascularization, was placed on veno-arterial extracorporeal membrane oxygenation (VA-ECMO). This was later transitioned to percutaneous right atrial to pulmonary artery right ventricular support, which led to subsequent recovery.

## Introduction

Transcatheter aortic valve replacement (TAVR) is an alternative to surgical aortic valve replacement (SAVR) in patients with high or intermediate surgical risk [1-3]. Recently, clinical trials have shown that TAVR is as good as SAVR among patients with severe aortic stenosis at low surgical risk [4]. The entire field of TAVR is evolving with the emergence of more experienced operators and a combination of better equipment, case selection, and imaging without having any deterioration of the valve structure over long-term follow-ups. Complications associated with TAVR include stroke, conduction abnormalities requiring a permanent pacemaker, vascular access complications, landing zone rupture, and coronary occlusion [5]. The purpose of this report is to describe a rare complication during TAVR valve deployment causing cardiogenic shock with initial stabilization on veno-arterial extracorporeal membrane oxygenation (VA-ECMO) and our strategy to transition to a right ventricular assist device (RVAD), which resulted in a successful recovery.

## Case presentation

A 71-year-old male patient was referred to the valve clinic for dyspnea on exertion attributed to severe aortic stenosis. He was found to be in the New York Heart Association (NYHA) Class III heart failure. His past medical history was significant for hypertension, dyslipidemia, and chronic obstructive pulmonary disease attributed to a remote 25-pack year smoking history. His surgical and family history was noncontributory to the current presentation. His physical examination was significant for 3/6 crescendo-decrescendo systolic murmur and pulsus parvus et tardus. His Katz frailty scale score was three.

**Figure 1:**
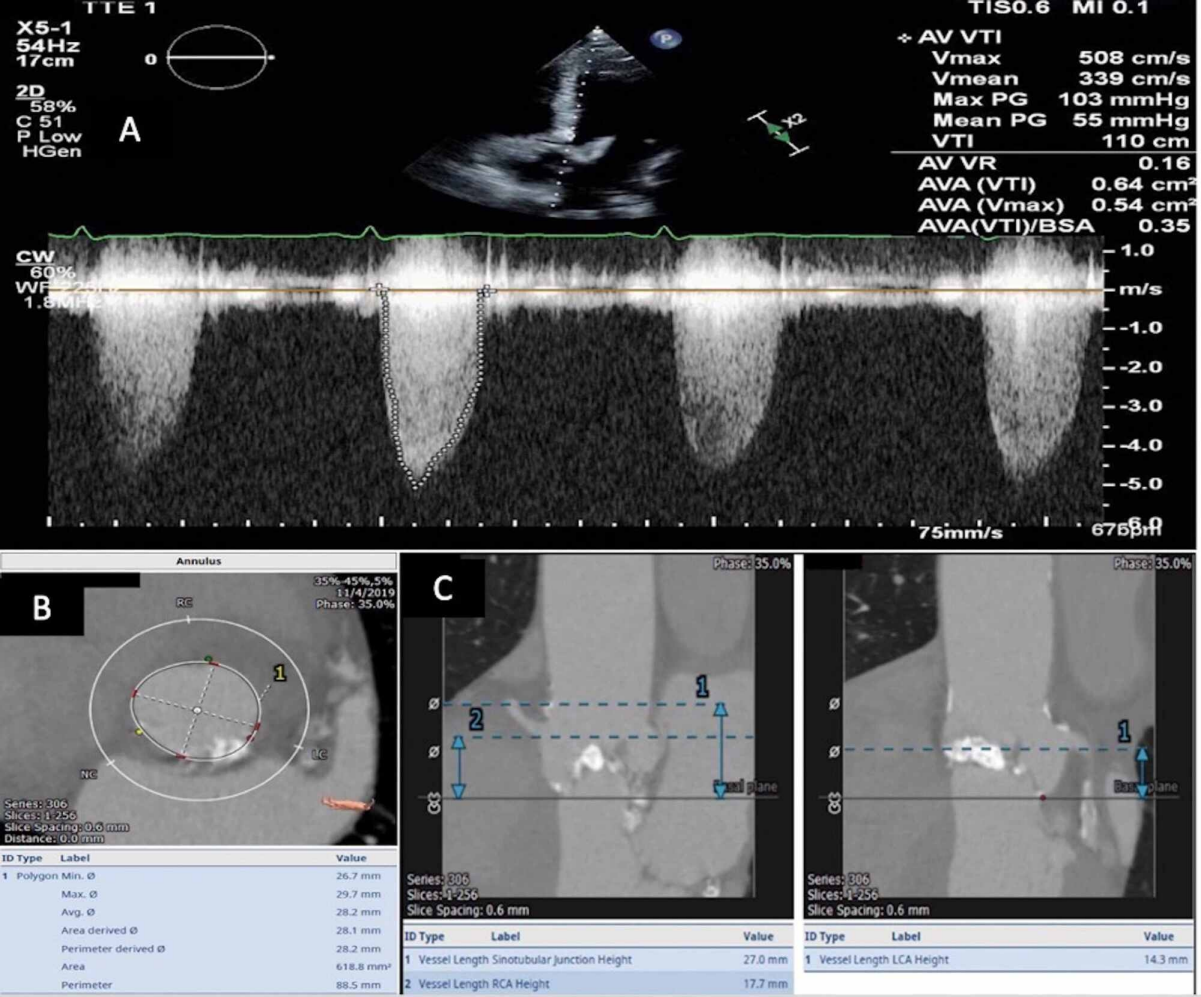
A: continuous wave doppler across the aortic valve; B: aortic annular dimensions; C: right and left coronary ostial heights from the aortic annulus

**Figure 2:**
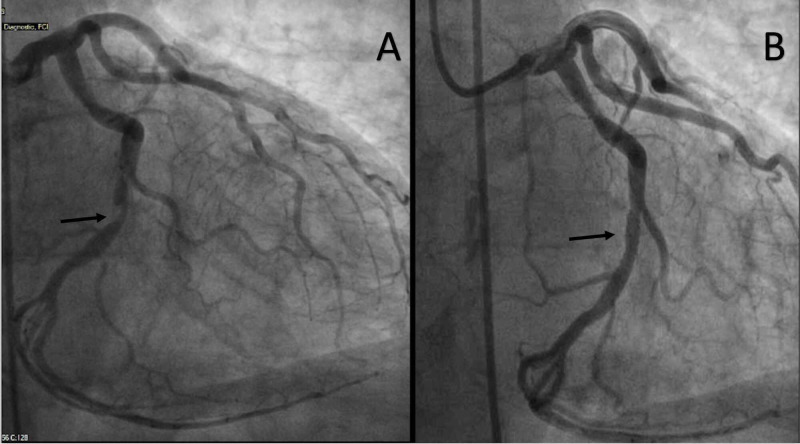
High-grade eccentric mid-left circumflex stenosis intervened on by an everolimus-eluting stent A: before the intervention; B: after the intervention

Echocardiogram performed prior to the referral had demonstrated an ejection fraction of 65%, aortic valve area of 0.64 cm^2^, peak velocity of 5.08 m/s, and mean gradient of 55 mmHg across the aortic valve (Figure 1A). He was evaluated by the Heart Team and was determined to have a Society of Thoracic Surgeons (STS) mortality score of 1.2% and to be a low risk for SAVR. Based on patient preference, TAVR was chosen over SAVR. A cardiac CT was performed, which demonstrated an annular area of 618.8 mm^2^, an annular perimeter of 88.5 mm, and the coronary heights to be 14.3 mm on the left and 17.7 mm on the right (Figure 1B, 1C). An ensuing diagnostic coronary angiogram was performed, which showed a high-grade eccentric mid-left circumflex stenosis, which was intervened upon by a 3.5 x 15 mm everolimus-eluting stent (Figure 2).

The right heart catheterization was unremarkable (mean right atrial pressure: 4 mmHg; pulmonary artery pressure: 32/13 mmHg with a mean of 20 mmHg; pulmonary capillary wedge pressure: 10 mmHg).

**Figure 3:**
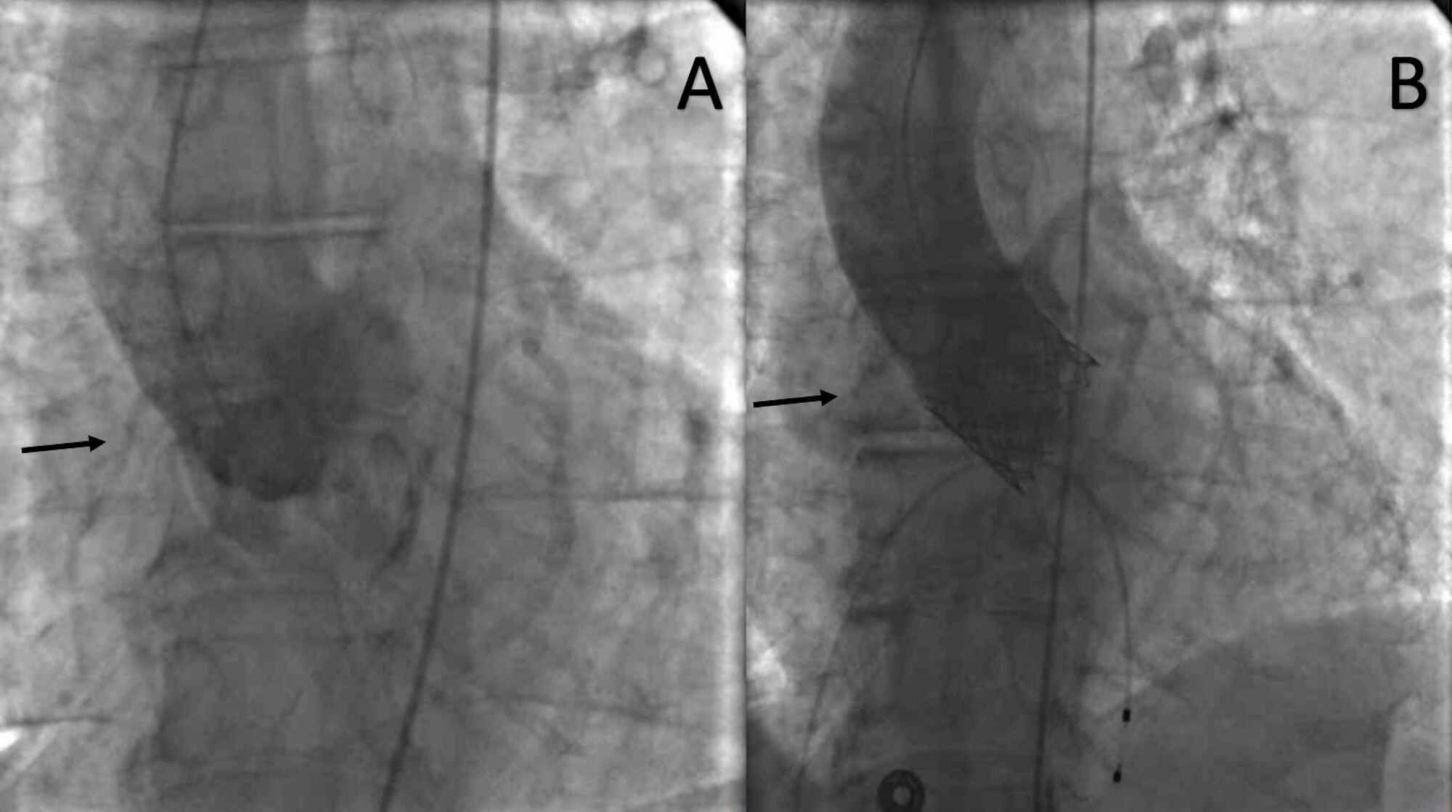
Aortic shot before and after valve deployment Small non-dominant right coronary artery can be seen in ‘A’, which appears to be occluded in ‘B’ post-intervention

As scheduled, the patient was brought in for a femoral access TAVR. The left femoral artery and venous were accessed with a 6 French (Fr.) sheath, and a 16 Fr. arterial access was obtained in the right femoral artery. A balloon aortic valvuloplasty was performed by a 25-mm Edwards standard balloon (Edwards Lifesciences, Irvine, CA). The planned 29-mm Edward Sapien 3 valve (Edwards Lifesciences, Irvine, CA) was successfully deployed under rapid pacing. At this point, the patient started complaining of chest pain. Immediately, an echocardiogram was obtained, which showed the left ventricular ejection fraction to be preserved with no pericardial effusion and no significant aortic regurgitation. However, the right ventricle appeared dilated. An aortic root shot was obtained, which demonstrated reduced opacification of the right coronary artery (RCA) (Figure 3).

**Figure 4:**
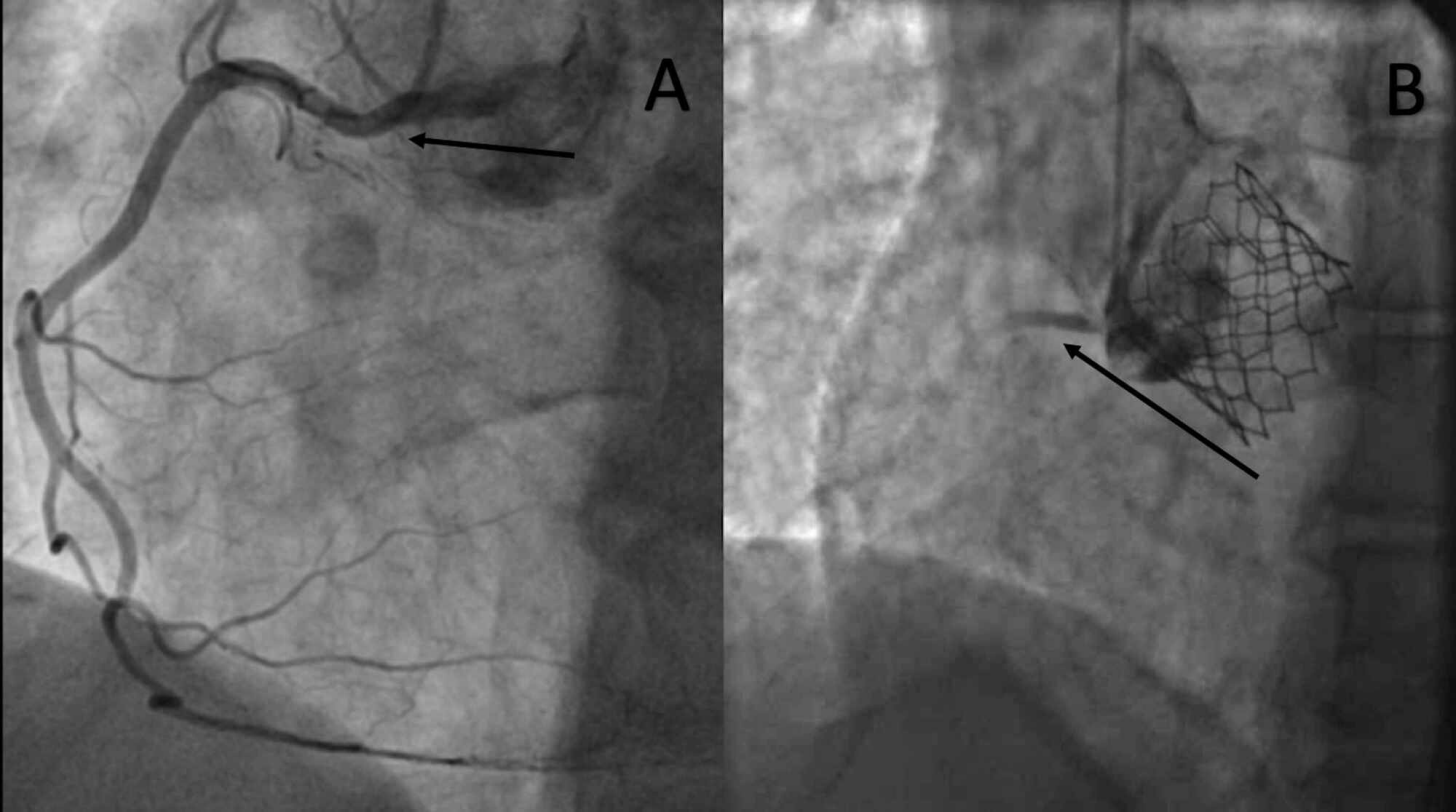
A: selective RCA angiography with JR catheter pre-TAVR; B: difficulty in cannulation due to ostial occlusion immediately post valve deployment RCA: right coronary artery; JR: Judkins Right; TAVR: transcatheter aortic valve replacement

Selective RCA angiography was attempted with multiple different guide catheters [Judkins Right (JR)4, JR3.5, AR1, AR2, and LIMA] but was unsuccessful (Figure 4), likely due to ostial occlusion, making cannulation challenging and unsuccessful. The patient continued to have hypotension despite ongoing volume resuscitation. An intra-aortic balloon pump (IABP) was placed at this time. A pulmonary artery catheter placement was being attempted when the patient had ventricular fibrillation. He was defibrillated twice with a direct current 250 J. Despite the return of circulation, the patient was hypotensive and in circulatory shock. Given the small RCA vessel, which was non-dominant, and the clinical state, the Heart Team chose ECMO over surgical revascularization. The patient was placed on ECMO with the right internal jugular (IJ) vein as venous access and the right femoral artery as the return cannula.

It was clear during the procedure that the patient had an acute right ventricular failure due to ostial RCA occlusion. However, instead of right ventricular support with either a microaxial pump or a right atrial to pulmonary arterial (RA-PA) centrifugal pump, the patient was placed on ECMO due to concerns of ventricular arrhythmia. The patient was then monitored in the intensive care unit where he did not have any further recurrence of ventricular arrhythmia. His left ventricular function was normal. During cannulation, he developed a femoral arterial tear necessitating vascular graft placement. He developed significant bleeding from the groin site requiring multiple transfusions. At this time, due to continued right ventricular failure and peripheral vascular complication with ECMO, plans were made to transition from ECMO to right ventricular support in the form of an RA-PA centrifugal pump the next day.

**Figure 5:**
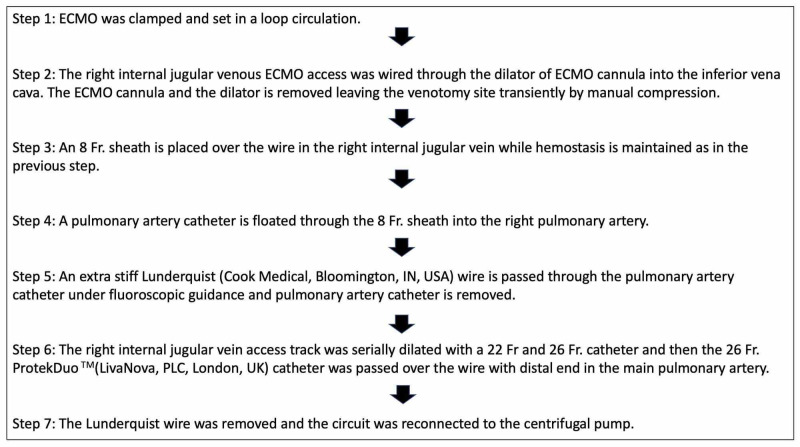
Transition steps from ECMO to Protek Duo® ECMO: extracorporeal membrane oxygenation

**Figure 6:**
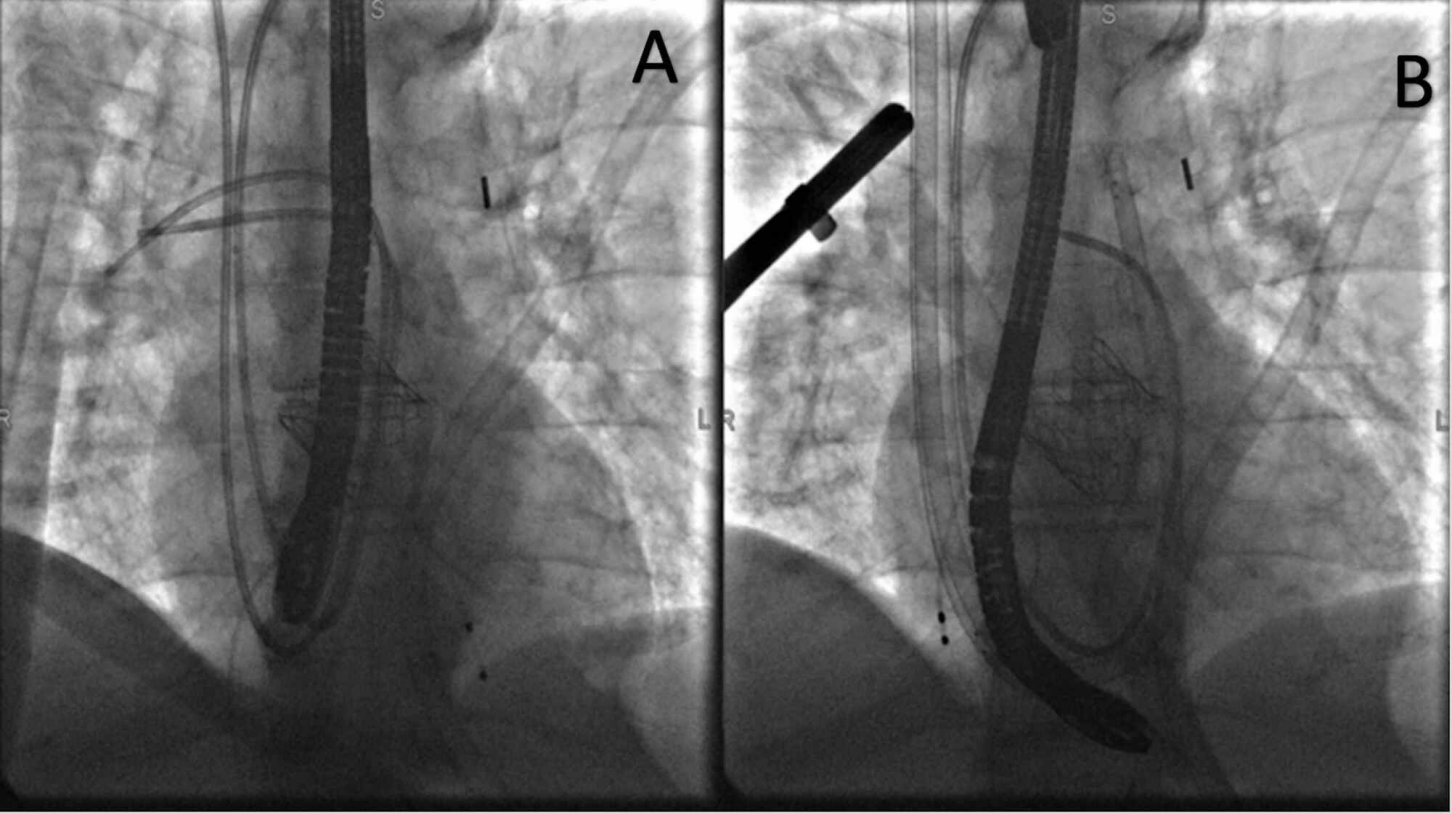
A: PA catheter in place with extra stiff Lunderquist wire, IABP, and a temporary pacemaker in place; B: Protek Duo was passed over the stiff wire with distal end reaching the main pulmonary artery PA: pulmonary artery; IABP: intra-aortic balloon pump

The ECMO access was in the right IJ where the access cannula went all the way into the inferior vena cava. The transition from this configuration to a cannula with the same access in the right IJ with the distal end in the main pulmonary artery was a challenge; this was because the patient was dependent on the ECMO for hemodynamic support. The transition steps were discussed and coordinated as seen in Figure 5 and Figure 6. Finally, the Lunderquist wire was removed and the circuit was reconnected to the centrifugal pump.

The patient remained clinically stable on Protek Duo (LivaNova PLC, London, UK), and his IABP was weaned off on day three. An echo-guided turndown study was done on day three, and the right ventricle demonstrated preserved basal and apical segments; however, mid-segment of the right ventricular free wall was akinetic. On day four, he was decannulated and was maintaining hemodynamics on inotropes and pressor support. He was weaned off pressor support and was eventually extubated on day eight. He went into complete heart block and received a permanent pacemaker on day 15. He was discharged on day 20 to cardiac rehabilitation. He was seen at the follow-up clinic with a repeat echocardiogram within two weeks of discharge, and it showed that the right ventricular size and systolic function had significantly improved when compared to previous studies.

## Discussion

The incidence of coronary obstruction has been reported in several TAVR trials to be less than 1% [6-9]. Baseline and procedural variables that are associated with coronary occlusion include older age, female sex, low coronary ostial height, aortic root size, size of the cusp, calcium burden and the location of calcium on the cusp, no previous coronary artery bypass grafting (CABG), and the use of a balloon-expandable valve and previous surgical aortic bioprosthesis [10]. Left coronary ostia (90%) was more commonly involved as compared to the right (10%) due to its anatomic proximity to the aortic valve. TAVR is usually discouraged when the ostial height is <12 mm and the sinus of the Valsalva diameter is <30 mm [11]. Special consideration is given for the bicuspid valve since leaflets are longer than in the tricuspid valve, which increases the chance of ostial occlusion despite being within safe margins [12].

Coronary obstruction during TAVR is a rare yet life-threatening complication with a 30-day mortality rate of around 40% [11]. coronary obstruction can occur immediately after valve deployment or can be delayed when the patient has left the operating room in a stable condition following successful TAVR. Delayed coronary occlusion (DCO) is further subdivided into two types: early (<seven days) and late (>seven days). The proposed mechanism behind early DCO includes continuing the expansion of the implanted valve, or dissection or hematoma causing the obstruction. In contrast, thrombus or valve stent endothelization may cause late DCO [13]. Most patients present with severe hypotension, and about half of them exhibit EKG changes, mainly ST-segment elevation, and more than one-third have ventricular arrhythmias.

The Heart Team approach is essential in the management of this complication. Acute coronary occlusion during TAVR results in profound hemodynamic collapse and placing the patient on VA-ECMO is crucial [14]. This is followed by direct stenting of the occlusion or repositioning of the TAVR prosthesis. If this fails, a surgical procedure is required. In high-risk cases, new prophylactic techniques including chimney snorkel and basilica have been implemented; however, the data regarding them is scarce [15,16].

## Conclusions

We described a series of unexpected and rare complications in a patient undergoing TAVR as a result of acute ostial RCA occlusion. We also described our strategy of transitioning from VA-ECMO to RA-PA RVAD and successful recovery. Our patient did have ostial RCA occlusion and it was presumed to be from heavily calcified cusps; however, since it was a non-dominant artery, the benefit of surgical revascularization of the right ventricular branch was minimal. It was crucial to place the patient on VA-ECMO due to electrical instability from ischemia caused by acute occlusion. Once the infarction was completed, VA-ECMO was transitioned to RA-PA centrifugal RVAD. In our case, the risk for ostial occlusion was not anticipated considering the distance from the aortic annulus, which was 17.7 mm for RCA. Hence, it is just not the distance of the coronary Ostia from the annulus or the aortic annular diameter that predicts the risk; other factors such as the length of the cusps and degree of calcification, leaflet thickness, sinus depth, and cephalad movement of leaflets also predict this catastrophic complication.
